# Early pretend play: a novel approach to understanding the development of anorexia nervosa

**DOI:** 10.1186/2050-2974-1-S1-O46

**Published:** 2013-11-14

**Authors:** Genevieve Pepin, Karen Stagnitti

**Affiliations:** 1 School of Health and Social Development, Deakin University, Australia

## 

Similarities between anorexia nervosa (AN) and autism spectrum disorders (ASD) such as poor cognitive flexibility, difficulties with set shifting, and impaired interpersonal functioning and social interactions have been identified in the literature. Evidence concerning persons with ASD established that these characteristics can be linked to poor pretend play in childhood and that improved pretend play ability increased cognitive flexibility and social competence. This study examines early play histories of persons with AN and their current flexible thinking abilities.

A mixed methods design using quantitative analysis of flexible thinking measures and retrospective interviews exploring early play histories is being implemented with twenty young people aged 13 to 17 years with AN and their primary carers.

This paper will present and critique the results of this study currently taking place in Victoria, Australia. Results will provide new information about AN from a perspective that has not been explored, contribute to the growing body of knowledge on the similarities between ASD and AN, inform professionals about how play behaviours may alert to or be early signs of some behaviours specific to AN, and potentially, provide guidelines for intervention development for these persons.

This abstract was presented in the **Anorexia Nervosa – Characteristics and Treatment** stream of the 2013 ANZAED Conference.

